# NAN-DETR: noising multi-anchor makes DETR better for object detection

**DOI:** 10.3389/fnbot.2024.1484088

**Published:** 2024-10-14

**Authors:** Zixin Huang, Xuesong Tao, Xinyuan Liu

**Affiliations:** School of Computer Science, Beijing Institute of Technology, Beijing, China

**Keywords:** object detection, transformer, multi-anchor, noising mechanism, CIoU

## Abstract

Object detection plays a crucial role in robotic vision, focusing on accurately identifying and localizing objects within images. However, many existing methods encounter limitations, particularly when it comes to effectively implementing a one-to-many matching strategy. To address these challenges, we propose NAN-DETR (Noising Multi-Anchor Detection Transformer), an innovative framework based on DETR (Detection Transformer). NAN-DETR introduces three key improvements to transformer-based object detection: a decoder-based multi-anchor strategy, a centralization noising mechanism, and the integration of Complete Intersection over Union (CIoU) loss. The multi-anchor strategy leverages multiple anchors per object, significantly enhancing detection accuracy by improving the one-to-many matching process. The centralization noising mechanism mitigates conflicts among anchors by injecting controlled noise into the detection boxes, thereby increasing the robustness of the model. Additionally, CIoU loss, which incorporates both aspect ratio and spatial distance in its calculations, results in more precise bounding box predictions compared to the conventional IoU loss. Although NAN-DETR may not drastically improve real-time processing capabilities, its exceptional performance positions it as a highly reliable solution for diverse object detection scenarios.

## 1 Introduction

Object detection remains a cornerstone task in robotic vision, with the primary objective of accurately identifying and localizing objects within an image. A variety of approaches have emerged over the years, including the influential R-CNN family and its subsequent variations (Ren et al., 2015; Zhang et al., 2020). For example, FoveaBox (Kong et al., 2020) offers an anchor-free detection framework leveraging a multi-level feature pyramid to achieve high-quality results across different scales. Meanwhile, Soft-NMS (Bodla et al., 2017) introduces an advanced non-maximum suppression technique that adjusts detection scores in densely populated scenes, thereby enhancing accuracy. Traditional methods generally rely on one-to-many label assignment strategies, where multiple predictions are mapped to each ground truth box, often through proposals, anchors, or window centers. Despite their successes, these methods tend to rely heavily on complex, manually designed components such as non-maximum suppression (NMS) and anchor generation, which can lead to inefficiencies and inherent limitations in adaptability.

The advent of the Detection Transformer (DETR) (Carion et al., 2020) marked a significant shift in the object detection landscape by redefining the task as a set prediction problem, thereby dispensing with the need for traditional components such as NMS and anchors. By utilizing a transformer-based encoder-decoder architecture (Vaswani et al., 2017) and employing a one-to-one matching strategy through the Hungarian algorithm (Kuhn, 1955), DETR enables direct end-to-end optimization, simplifying the detection process. However, despite these innovations, DETR's adoption has been limited by issues such as slow convergence and the inherent challenges of its one-to-one matching strategy, which often leads to sparse supervision signals during training.

To mitigate the issues inherent in the original DETR framework, various enhancements have been developed over time. For instance, REGO (Chen Z. et al., 2022) enhances small object detection through optimized feature representation for specific regions. Salience-DETR (Hou et al., 2024) increases accuracy by emphasizing salient objects in images. Additionally, SMCA (Gao et al., 2021) employs a spatially modulated cross-attention mechanism to refine localization, and Sparse-DETR (Roh et al., 2022) introduces a sparse sampling strategy to reduce computational load, making it more suitable for real-time applications. Methods like UP-DETR (Dai et al., 2022) leverage unsupervised pre-training to improve performance in data-scarce environments, and WB-DETR (Liu F. et al., 2021) simplifies detection by removing the CNN backbone, relying instead on a pure Transformer-based architecture. Dynamic DETR (Dai et al., 2021) enhances flexibility through dynamic attention mechanisms, and Efficient DETR (Yao et al., 2021) reduces model complexity by optimizing resource usage. Together, these methods contribute to refining the DETR architecture by enhancing training efficiency, detection accuracy, and adaptability across diverse object detection tasks.

Recent advances demonstrate the effectiveness of enhancing feature learning and improving detection accuracy for boosting object detection performance. Co-DETR (Zong et al., 2023) decouples object query assignments and uses auxiliary queries for broader feature capture, while Group DETR (Chen et al., 2023) and NMS DETR (Ouyang-Zhang et al., 2022) employ one-to-many label assignments, with the latter integrating non-maximum suppression to refine outcomes. DN-DETR (Li et al., 2022) introduces denoising to stabilize training, a concept further optimized by DINO's (Zhang et al., 2023) contrastive learning approach. Additionally, in real-time detection, DIoU and CIoU losses (Zheng et al., 2020) have emerged, improving bounding box accuracy by addressing limitations in traditional IoU metrics through enhanced convergence speed and regression precision.

Previous methods have struggled to achieve effective one-to-many matching, while NAN-DETR (Noising multi-ANchor DEtection TRansformer) addresses these challenges through a series of novel improvements. The architecture of NAN-DETR consists of a backbone network, a multi-layer transformer encoder, several multi-layer transformer decoders, and multiple prediction heads. A key innovation is the multi-anchor strategy based on decoders, where multiple independent decoders refine the initial anchors generated by the encoder, thereby improving detection accuracy. Additionally, the introduction of a concentrated noise mechanism in the decoders minimizes conflicts between anchor boxes, further enhancing the robustness. Similar to DETR, the matching process employs the “Complete Intersection over Union” (CIoU) loss function to enhance anchor box similarity and optimize detection results. The combination of these innovative techniques significantly improves object detection accuracy, particularly in terms of Average Precision (AP) for objects of various sizes, distinguishing NAN-DETR from other DETR variants.

Our contributions can be summarized as follows:

- We present a new end-to-end DETR-type model with a centralization noising multi-anchor strategy, achieving high-accuracy object detection.
- We propose the Decoder-based multi-anchor strategy to enhance object detection accuracy and the centralization noising mechanism to reduce conflicts between different anchors. In addition, we employ the complete intersection over union (CIoU) loss to improve the precise measurement of similarity between anchors.
- We validate the effectiveness of NAN-DETR through comprehensive experiments on the COCO dataset, where our model, using ResNet-50 as the backbone, achieves an average precision (AP) of 50.1%, outperforming existing state-of-the-art methods.

The structure of this paper is as follows: In Section 2, we review the existing literature on object detection, focusing on the progress made in set matching, anchor-based techniques, and label assignment strategies. Section 3 delves into the fundamental aspects of the DETR framework and details the key innovations introduced in NAN-DETR, such as the decoder-based multi-anchor strategy and the centralization noising mechanism. Section 4 presents the experimental results, showcasing how NAN-DETR outperforms other DETR variants on the COCO dataset in terms of performance. Finally, Section 5 concludes the paper by underscoring the improvements NAN-DETR brings to detection accuracy and offering suggestions for future research.

## 2 Related works

This section reviews key developments in transformer-based object detection, particularly focusing on the DETR framework and various improvement strategies.

### 2.1 One-to-one set matching

DETR (Carion et al., 2020) introduced a significant shift in object detection by framing the task as a set prediction problem, utilizing a transformer architecture. This method employs a one-to-one matching strategy based on the Hungarian algorithm, enabling direct end-to-end training without the need for conventional components like non-maximum suppression (NMS). However, this one-to-one matching approach often results in sparse supervision signals and slower convergence rates during training, which can limit its effectiveness. To address these challenges, DN-DETR (Li et al., 2022) incorporated a denoising technique during the training process, which helps stabilize the matching process and speeds up convergence. By introducing noise into the training queries, DN-DETR mitigates the issues caused by sparse positive samples. Furthermore, Conditional DETR (Meng et al., 2021; Chen X. et al., 2022) enhances this approach by refining the query mechanism, leading to improved efficiency in model training and faster convergence, ultimately boosting detection accuracy.

### 2.2 Anchor matching

Although DETR initially removed the reliance on anchors, subsequent studies have shown that reintroducing anchors can significantly enhance performance. Approaches such as Anchor DETR (Wang et al., 2022) and DAB-DETR (Liu S. et al., 2022) reintegrate anchor boxes within the DETR framework, which not only makes the query process more interpretable but also accelerates convergence by narrowing the search space. By anchoring queries closer to likely object locations, these methods reduce computational complexity and improve overall model efficiency. DINO (Zhang et al., 2023) further refines this anchor-based strategy by integrating advanced denoising methods and contrastive learning, particularly enhancing detection accuracy in more complex scenarios. These developments highlight the effectiveness of merging traditional object detection techniques with transformer-based models, offering a pathway to superior detection performance.

### 2.3 One-to-many label assignment

Traditional object detection methods, including those in the R-CNN family (Ren et al., 2015; Zhang et al., 2020), typically utilize a one-to-many label assignment strategy, where multiple predictions correspond to each ground truth box. This concept has been effectively adapted into transformer-based models. For example, Group-DETR (Chen et al., 2023) employs a group-wise one-to-many assignment, allowing multiple queries to align with each ground truth box. This strategy strengthens the model's feature learning and attention mechanisms, resulting in improved detection performance. Co-DETR (Zong et al., 2023) further expands on this by incorporating flexible assignments through auxiliary heads like ATSS and Faster R-CNN, which enhance supervision and significantly boost accuracy, especially in densely populated scenes.

### 2.4 IoU

Intersection over Union (IoU) is a critical metric in computer vision, extensively used to assess the accuracy of object detection and segmentation models by measuring the overlap between predicted and ground truth bounding boxes. Although IoU became widely recognized through its application in the R-CNN framework (Girshick et al., 2014), it presents challenges, particularly when dealing with non-overlapping boxes. To overcome these limitations, Generalized IoU (GIoU) (Rezatofighi et al., 2019) was introduced, adding the concept of the smallest enclosing box to provide a more holistic measure. Further improvements include Distance-IoU (DIoU) and Complete-IoU (CIoU) (Zheng et al., 2020), which factor in the distance between box centers and aspect ratio, respectively, enhancing localization accuracy and convergence speed.

## 3 Methodology

### 3.1 Model overview

**Figure 1:**
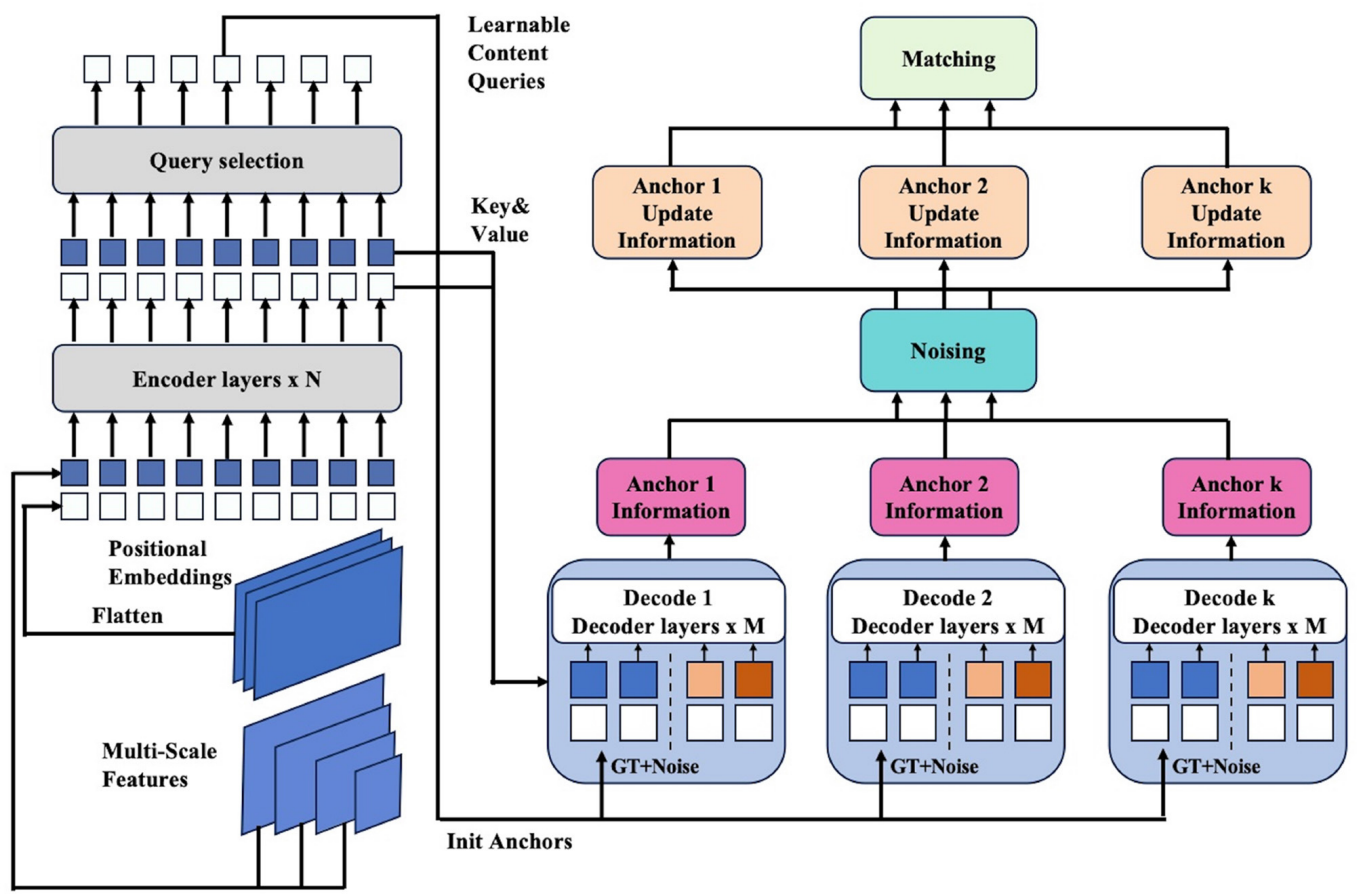
Framework of the proposed NAN-DETR. The enhancements primarily focus on the Transformer decoder. We use *k* decoders to acquire more anchors and reduce the conflicts between multi-anchor by centralization noising mechanism. Finally, we use CIoU loss to calculate the loss function of boxes in matching.

NAN-DETR enhances the DETR (Carion et al., 2020) framework with several key innovations aimed at boosting detection accuracy. The architecture includes a backbone network, a Transformer encoder, multiple Transformer decoders, and prediction heads that output the final detection results as Figure 1. The process starts by feeding the image into a backbone such as ResNet (He et al., 2016) or Swin-Transformer (Liu Z. et al., 2021, 2022), which extracts global features. These features, combined with positional embeddings to capture spatial relationships, are then processed by the Transformer encoder, dividing the image into multiple regions (queries). Details of the image feature extraction process can be obtained in Section 3.2. Each query is used to generate an initial anchor box through a neural network. These anchor boxes are then locally refined by *k* independent decoders to better detect the object. This strategy is called the decoder-based multi-anchor strategy, whose details are available in Section 3.3. To reduce conflicts between multiple anchor boxes, they are perturbed after being calculated, which is called as centralization noising mechanism presented in Section 3.4. Finally, the matching process is similar to DETR, but with CIoU (Zheng et al., 2020) introduced to improve the precise measurement of similarity between anchors and optimize the detection results, which is described in Section 3.5.

### 3.2 Image feature extraction

Given an image, we can obtain the visual feature knowledge through a visual backbone. In order to acquire different scale image information, we use multi-scale detection to extract multi-scale visual features. Meanwhile, as the position relationships between different regions in the image are highly important, we introduce position embedding to ensure that the position information of different regions can be captured by the model. The process of image feature extraction is as follows:


(1) $$\begin{array}{l} \text{v}=B\left(\text{x}\right)+{\text{v}}_{\text{pos}} \end{array}$$

where **v** represents the input of the Transformer encoder, *B* indicates the backbone, such as ResNet-50 (He et al., 2016) or Swin Transformer (Liu Z. et al., 2021, 2022) architecture, and **v**_pos_ denotes the sinusoidal position embedding. ResNet-50 uses convolution and residual connections, excelling at extracting local features with high computational efficiency, while Swin Transformer is based on self-attention, capturing both global and local information, making it suitable for complex vision tasks but with higher computational cost.

Next, we add the image features of the position embedding into the Transformer Encoder for attention interaction to get image features. Then, multiple query anchor frames for each object in the image are obtained as input to the Transformer decoder in the full connection layer.

### 3.3 Decoder-based multi-anchor strategy

The decoder-based multi-anchor strategy can alleviate the limitations of the initial DETR framework. In the DETR architecture, the encoder functions similarly to a standard Transformer encoder, producing abstract information that effectively divides the image into several regions, referred to as queries. To enhance object detection within these query regions, we introduce a neural network layer that generates four-dimensional vectors corresponding to anchor boxes (Wang et al., 2022; Zhang et al., 2023). These vectors, considered as initial anchor boxes, provide preliminary spatial information that indicates potential object locations.

However, a single anchor box often fails to adequately represent larger objects, leading to convergence challenges and difficulties in model training. While some ideas have been proposed in prior works, such as Group DETR (Chen et al., 2023) and Co-DETR (Zong et al., 2023), their research has all introduced a one-to-many matching approach. But our implementation diverges in its approach and takes some advantages over them. Group DETR employs arbitrary grouping, which does not fully leverage the information from the encoder, whereas Co-DETR relies heavily on auxiliary heads, leading to redundancy.

**Figure 2:**
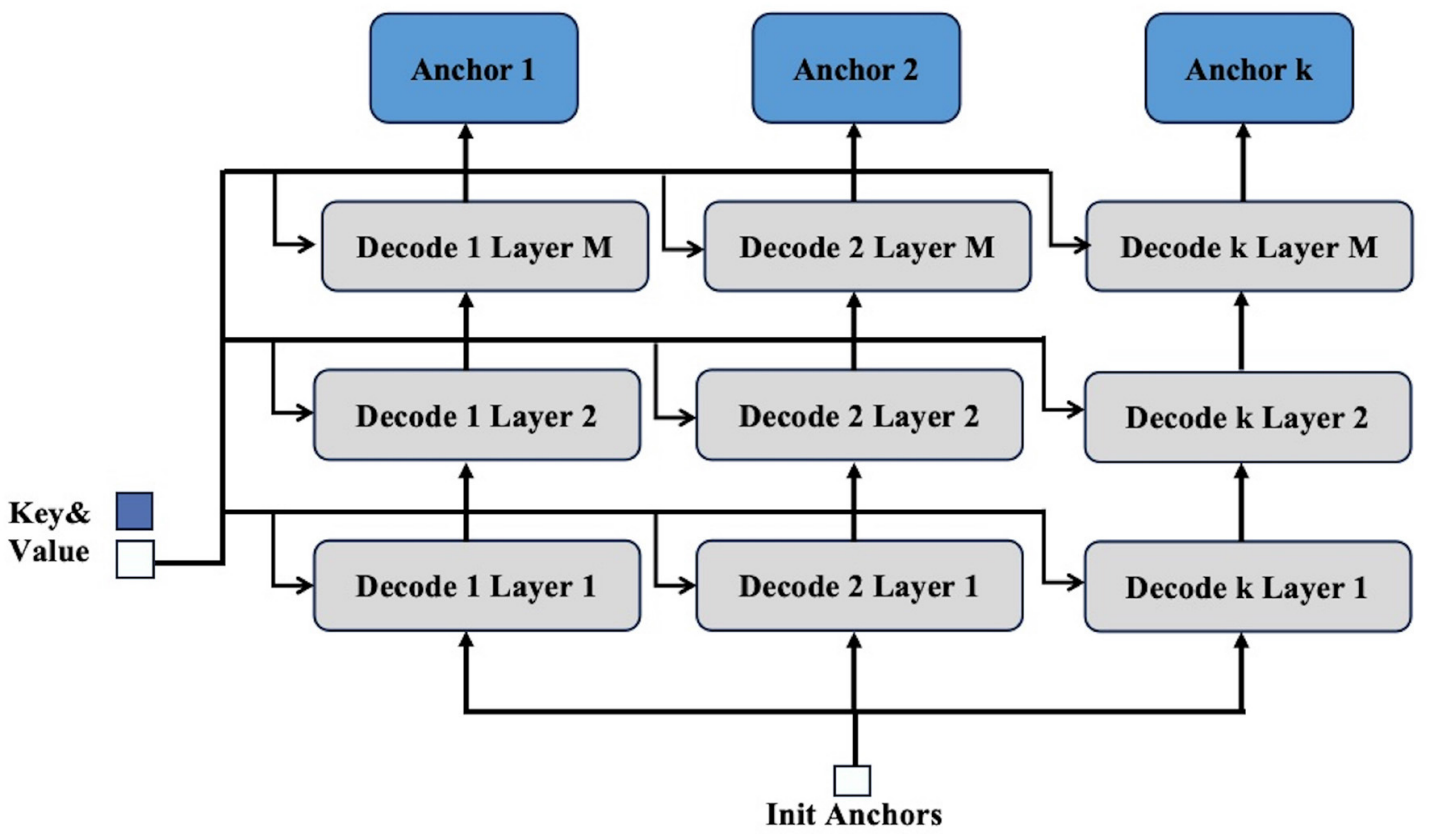
Details of decoder-based multi-anchor strategy. The process of this strategy mainly involves first creating *k* decoders. The initial anchor produced by the encoder is then sent to each decoder, while the key and value outputs from the encoder are provided to each layer of the decoders for processing. This allows a single query to obtain *k* anchors, thereby achieving a one-to-many effect.

To address these issues, our proposed strategy utilizes multiple decoders, denoted as *k* decoders, to process each query independently as Figure 2. Each decoder refines the initial anchor box, resulting in multiple predicted positions for anchor boxes. This one-to-many assignment effectively increases the likelihood of accurately capturing objects of varying scales within the image. Compared with Group DETR and Co-DETR, our strategy circumvents these limitations by refining anchor boxes through a more targeted and efficient process, ensuring better utilization of the encoder's output.

### 3.4 Centralization noising mechanism

Upon generating multiple anchor boxes for each query, a significant challenge emerges due to potential conflicts between the outputs of auxiliary heads, as noted in the Co-DETR study (Zong et al., 2023). For instance, consider a scenario where an object, such as a square, is initially represented by four anchor boxes centered within it. If these anchors are perturbed randomly, they may shift toward the four corners of the square. This perturbation can cause the anchors to lose sight of the object as a whole, with some potentially deviating completely from the square's boundaries. This phenomenon illustrates that different anchors may capture disparate and sometimes conflicting information about the same object, particularly when dealing with large objects. If these conflicts among the anchors are not effectively managed, the cumulative information they provide could become inconsistent or misleading, undermining the overall detection accuracy. This issue is akin to the aforementioned square example, where the anchors' divergence leads to incomplete or erroneous object representation.

**Figure 3:**
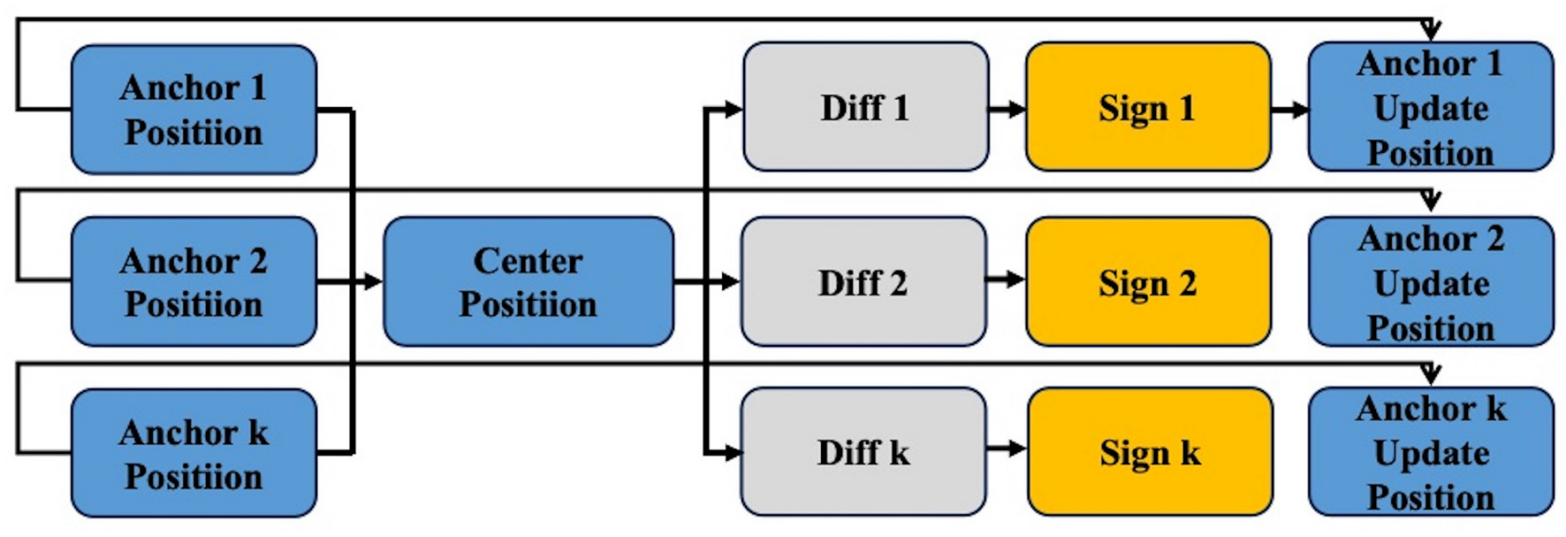
Details of centralization noising mechanism. The main process of the centralization noising mechanism first involves finding the centroid of all obtained anchors. Then, the difference (diff) for each anchor is calculated to determine the direction and magnitude of movement (noise). Finally, the updated anchors are derived by combining the sign with the original anchors.

To avoid this problem, we perturb these *k* anchor boxes. After calculating the center of these *k* anchor boxes, we apply random noise to them, causing them to move a certain distance toward the center. This step minimizes conflicts and merges the detection information from multiple boxes. As a result, we obtain *k* anchor boxes that influence each other and incorporate the possibilities of transformations. These *k*×query number anchor boxes will then be used for matching, as Figure 3, following the same matching process as in DETR.

Specifically, the centralization noising process is defined as follows: Given a set of detection boxes {(*x*_*i*_, *y*_*i*_, *u*_*i*_, *d*_*i*_)} along with$\left(\frac{x_{i}+u_{i}}{2},\frac{y_{i}+d_{i}}{2}\right)$ as their center, there exists a line *Ax*+*By*+*C* = 0, where both $\left(\frac{x_{i}+u_{i}}{2},\frac{y_{i}+d_{i}}{2}\right)$ and $\left(\frac{\sum_{i=1}^{k}x_{i}+u_{i}}{2k},\frac{\sum_{i=1}^{k}y_{i}+d_{i}}{2k}\right)$ lie on this line, thus we can denote *A, B, C* as


(2) $$\begin{array}{l} A=\frac{\sum_{i=1}^{k}y_{i}+d_{i}}{2k}-\frac{y_{i}+d_{i}}{2} \end{array}$$

(3) $$\begin{array}{l} B=\frac{\sum_{i=1}^{k}x_{i}+u_{i}}{2k}-\frac{x_{i}+u_{i}}{2} \end{array}$$

(4) $$\begin{array}{l} C=\frac{\left(\sum_{i=1}^{k}x_{i}+u_{i}\right)\left(y_{i}+d_{i}\right)}{4k}-\frac{\left(\sum_{i=1}^{k}y_{i}+d_{i}\right)\left(x_{i}+u_{i}\right)}{4k}. \end{array}$$

Moreover, the random noise is set to Δ~*N*(0, σ(*w*+*h*)), where *w, h* denote the width and height of the box respectively, and σ denotes the standard derivation. Then the actual center of the detection box becomes


(5) $$\begin{array}{l} {\text{Center}}_{i}=\left(\frac{x_{i}+u_{i}}{2}+\text{sign}\left(i\right)B\text{Δ},\frac{y_{i}+d_{i}}{2}-\text{sign}\left(i\right)A\text{Δ}\right), \end{array}$$

where $\text{sign}\left(i\right)=\left\{ \begin{array}{cc} 1 & \left(\frac{\overset{k}{\underset{i=1}{\sum }}x_{i}+u_{i}}{2k}-\frac{x_{i}+u_{i}}{2}\right)B\Delta <0\\ -1 & \text{otherwise} \end{array}\right..$

### 3.5 Complete Intersection over Union (CIoU) loss

Intersection over Union (IoU) is a widely used metric in object detection, primarily measuring the overlap between predicted and ground truth bounding boxes. However, IoU has notable limitations, such as failing to provide useful gradient information when boxes do not overlap and not fully accounting for variations in overlap due to translation or rotation. To address these shortcomings, we employ Complete Intersection over Union (CIoU) loss (Zheng et al., 2020), which has shown success in models such as YOLO (Zhao et al., 2024; Redmon et al., 2016). CIoU extends the basic IoU by incorporating the aspect ratio and the distance between the centers of bounding boxes, providing a more comprehensive assessment of similarity. In NAN-DETR, we replace the traditional IoU with CIoU, leveraging its ability to improve the precision of bounding box predictions during training, which is particularly beneficial for high-precision object detection tasks.

Specifically, The CIoU loss is defined as:


(6) $$\begin{array}{l} L_{\text{CIoU}}=1-\text{IoU}\left(b,b_{gt}\right)+\frac{\rho^{2}\left(b,b_{gt}\right)}{c^{2}}+\alpha v \end{array}$$

where:

- IoU(*b, b*_*gt*_) is the Intersection over Union between the predicted box *b* and the ground truth box *b*_*gt*_,
- ρ(*b, b*_*gt*_) is the Euclidean distance between the centers of the two boxes,
- *c* is the diagonal length of the smallest enclosing box for both boxes,
- α is a weight that balances the aspect ratio consistency *v*, which measures the consistency of the aspect ratios of the predicted box and the ground truth box.

NAN-DETR utilizes a composite loss function that integrates the Hungarian matching loss for query-object assignment and the CIoU loss for bounding box regression. The overall loss L is given by:


(7) $$\begin{array}{l} L=\lambda_{1}L_{\text{Hungarian}}+\lambda_{2}L_{\text{Box}}+\lambda_{3}L_{\text{CIoU}} \end{array}$$

where $L_{\text{Hungarian}}$ is the Hungarian matching loss (Carion et al., 2020; Kuhn, 1955), $L_{\text{Box}}$ denotes the ℓ_1_-distance of predicted box and matched box, and λ_1_, λ_2_, λ_3_ are hyperparameters that balance the two components of the loss function.

## 4 Experiments

### 4.1 Setup

#### 4.1.1 Datasets and evaluation metrics

To assess the performance of NAN-DETR, we conducted evaluations using the COCO dataset (Lin et al., 2015), a comprehensive benchmark widely adopted in object detection research. The dataset encompasses 80 object categories and over 200,000 labeled images. The val subset represents the detection results that we report. The main evaluation metric is Average Precision (AP), which measures the area under the precision-recall curve averaged across all categories. Specific metrics, such as AP_50_ and AP_75_, correspond to the Average Precision when the IoU threshold is 0.5 and 0.75, respectively. In addition, the AP_*S*_, AP_*M*_, and AP_*L*_ metrics evaluate the performance for different object sizes (small, medium, and large), providing a deep insight into the ability of NAN-DETR to tackle different detection challenges.

#### 4.1.2 Implementation details

NAN-DETR is implemented using PyTorch and trained on a setup comprising 8 NVIDIA A100 GPUs. We utilize the AdamW optimizer with a base learning rate of 10^−4^ and a lower learning rate of 10^−5^ for the backbone. The model training involves clipping the maximum gradient norm at 0.1, with a positional encoding temperature set to 20. Both the encoder and decoder are composed of 6 layers, each with a feedforward dimension of 2048 and a hidden dimension of 256, without applying dropout. The model operates with 8 attention heads and processes 900 queries, each comprising 4 points in both the encoder and decoder. ReLU serves as the activation function, and FrozenBatchNorm2d is used for batch normalization. The model's cost settings include values of 2.0 for class prediction, 5.0 for bounding boxes, and 2.0 for CIoU. We set the classification loss coefficient to 1.0, with bounding box and CIoU loss coefficients at 5.0 and 2.0, respectively. Additionally, the focal loss alpha parameter is 0.25, and the noise parameter σ is fixed at 0.05. These hyperparameters were fine-tuned based on extensive experimentation to optimize model performance.

### 4.2 Baseline methods

We compare NAN-DETR with various state-of-the-art DETR variants:

- Conditional-DETR (Meng et al., 2021; Chen X. et al., 2022): Conditional-DETR introduces key methodological improvements over DETR, primarily by enhancing the query mechanism, decoder module, and matching strategy.
- Anchor-DETR (Wang et al., 2022): Anchor-DETR brings significant enhancements to the original DETR by incorporating object queries that are designed around anchor points, a concept widely utilized in CNN-based detectors.
- DAB-DETR (Liu S. et al., 2022): DAB-DETR enhances the original DETR by introducing dynamic anchor boxes as queries, with both their position and size being dynamically adjusted layer by layer.
- AdaMixer (Gao et al., 2022): Compared with DETR, AdaMixer features adaptive 3D feature sampling, where queries dynamically sample features across different spatial and scale dimensions.
- Deformable-DETR (Zhu et al., 2021): Deformable-DETR advances the original DETR by incorporating a deformable attention module that focuses on a limited set of key sampling points near a reference point, rather than considering all spatial locations.
- DN-Deformable-DETR (Li et al., 2022): DN-Deformable-DETR enhances the original DETR by implementing a denoising training technique that stabilizes the bipartite graph matching process, which is often unstable during the early stages of training. This technique involves inputting noisy ground truth bounding boxes into the transformer decoder and training the model to accurately reconstruct the original boxes, thereby speeding up convergence and boosting overall model performance.
- H-Deformable-DETR (Jia et al., 2023): H-Deformable-DETR enhances the original DETR by introducing a hybrid matching strategy, which integrates one-to-one matching with an additional one-to-many matching branch during the training process.
- DINO-Deformable-DETR (Zhang et al., 2023): DINO improves upon DETR by introducing several key advancements: a contrastive denoising training method to handle noisy data, a mixed query selection strategy to better initialize queries, and a “look forward twice” scheme that enhances the box prediction process by refining parameters from both the current and subsequent layers.
- Co-Deformable-DETR (Zong et al., 2023): Co-Deformable-DETR improves upon DETR by introducing a collaborative hybrid assignment training scheme with auxiliary heads that combines one-to-one and one-to-many label assignments.

### 4.3 Main results

**Figure 4:**
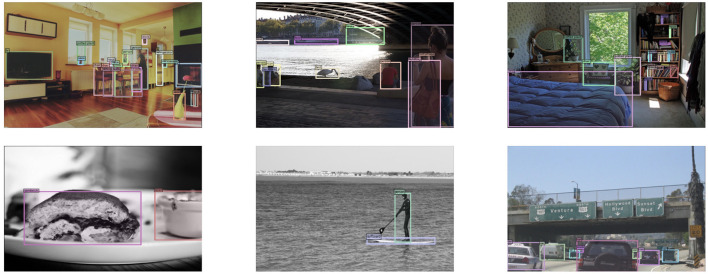
Visualizations of COCO dataset by NAN-DETR with ResNet-50.

Our method performs well on the COCO dataset, and the specific visualization results are shown in Figure 4.

#### 4.3.1 ResNet-50 backbone

**Figure 5:**
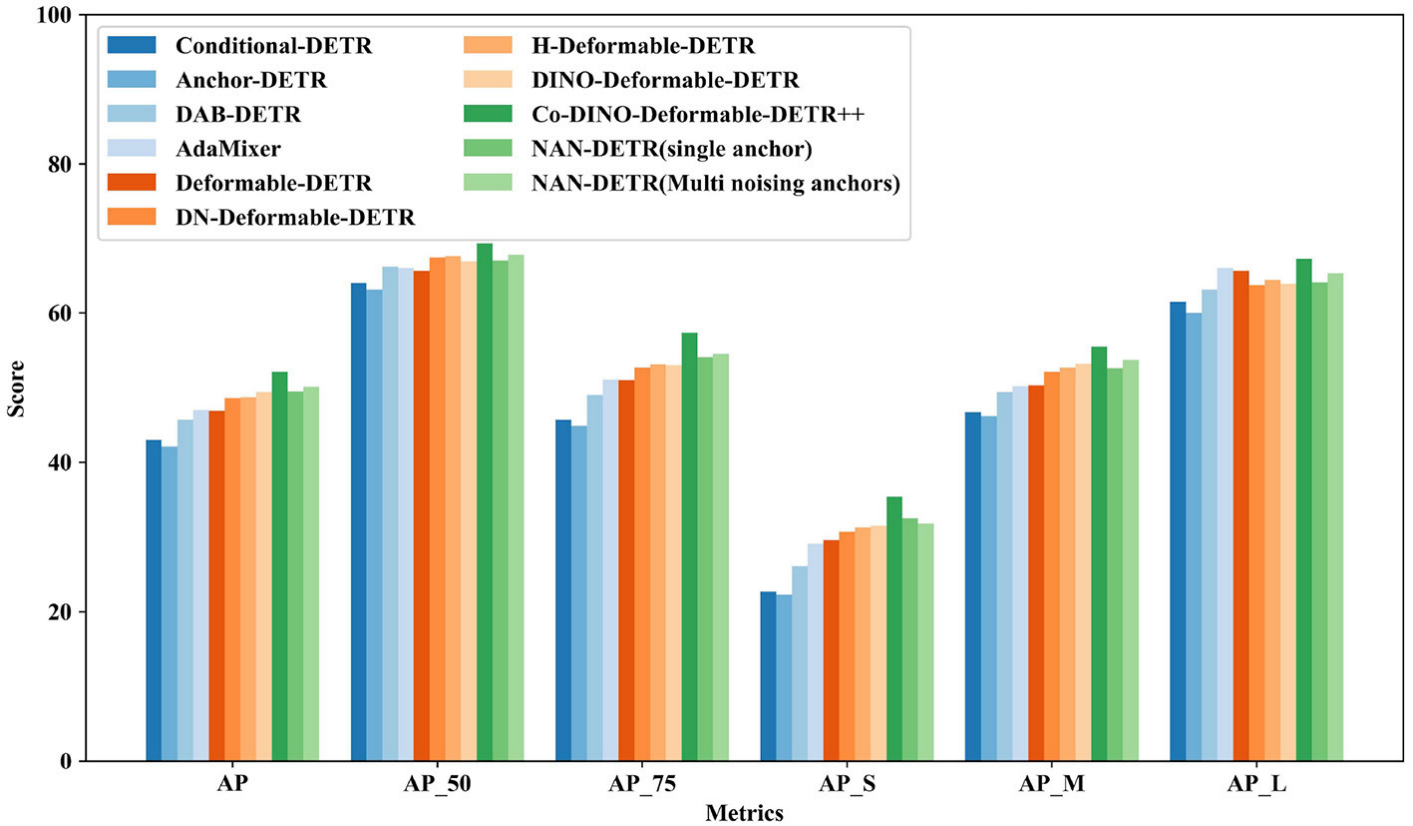
Comparison to the baselines DETR variants on COCO val with ResNet-50.

The performance of various DETR variants using the ResNet-50 backbone in Table 1 and Figure 5 demonstrates significant differences in object detection capabilities. Methods such as Conditional-DETR (Meng et al., 2021; Chen X. et al., 2022) and Anchor-DETR (Wang et al., 2022), which reintroduce anchor boxes and conditional queries, show moderate performance with AP scores of 43.0% and 42.1%, respectively. These methods improve query interpretability but still lag behind more advanced models. DAB-DETR (Liu S. et al., 2022) and AdaMixer (Gao et al., 2022), incorporating dynamic anchor boxes and adaptive mixing strategies, achieve better AP scores of 45.7% and 47.0%, respectively, showing enhanced query formulation and faster convergence.

**Table 1:** Comparison to the baselines on COCO val with ResNet-50.

**Method**	**Backbone**	**Multi-scale**	**#query**	**#epochs**	**AP↑**	**AP_50_↑**	**AP_75_↑**	**AP_*S*_↑**	**AP_*M*_↑**	**AP_*L*_↑**
Conditional-DETR; Meng et al. (2021)	R50	✗	300	108	43.0	64.0	45.7	22.7	46.7	61.5
Anchor-DETR; Wang et al. (2022)	R50	✗	300	50	42.1	63.1	44.9	22.3	46.2	60.0
DAB-DETR; Liu S. et al. (2022)	R50	✗	900	50	45.7	66.2	49.0	26.1	49.4	63.1
AdaMixer; Gao et al. (2022)	R50	✓	300	36	47.0	66.0	51.1	30.1	50.2	61.8
Deformable-DETR; Zhu et al. (2021)	R50	✓	300	50	46.9	65.6	51.0	29.6	50.1	61.6
DN-Deformable-DETR; Li et al. (2022)	R50	✓	300	50	48.6	67.4	52.7	31.0	52.0	63.7
H-Deformable-DETR; Jia et al. (2023)	R50	✓	300	12	48.7	66.4	52.9	31.2	51.5	63.5
DINO-Deformable-DETR; Zhang et al. (2023)	R50	✓	900	12	49.4	66.9	53.8	32.3	52.5	63.9
Co-Deformable-DETR; Zong et al. (2023)	R50	✓	300	12	49.5	67.6	54.3	32.4	52.7	63.7
NAN-DETR(CIoU, single anchor)	R50	✓	900	12	49.5	67.0	54.1	**32.5**	52.6	64.1
NAN-DETR(CIoU, Multi-noising anchors)	R50	✓	900	12	**50.1**	**67.8**	**54.5**	31.8	**53.7**	**65.3**

Bolded and underlined fonts indicate the first two best values.

More advanced methods like DN-Deformable-DETR (Li et al., 2022) and H-Deformable-DETR (Jia et al., 2023) leverage deformable attention modules and denoising techniques, resulting in higher AP scores of 48.6% and 48.7%, respectively. DINO-Deformable-DETR (Zhang et al., 2023) further improves upon these techniques, achieving an AP of 49.4%. Co-Deformable-DETR (Zong et al., 2023) also demonstrates strong performance with an AP of 49.5%, showcasing the effectiveness of collaborative hybrid assignments and the importance of detection heads.

However, the top performer in this category is NAN-DETR with multi-noising anchors, achieving an impressive AP of 50.1%. This method outperforms all other DETR variants, demonstrating significant improvements in detection precision. The CIoU loss also enhances bounding box predictions, contributing to higher AP_75_ scores. As shown in Figure 7, while there is a slight decrease in performance for small objects, NAN-DETR consistently excels in detecting medium and large objects, making it a versatile and robust solution for various object detection tasks to resolve multiple anchors conflicts. From the results of AP_*S*_ and AP_*L*_, the centralization noising mechanism performs well and indeed achieves excellent results in large object detection.

#### 4.3.2 Swin backbone

**Figure 6:**
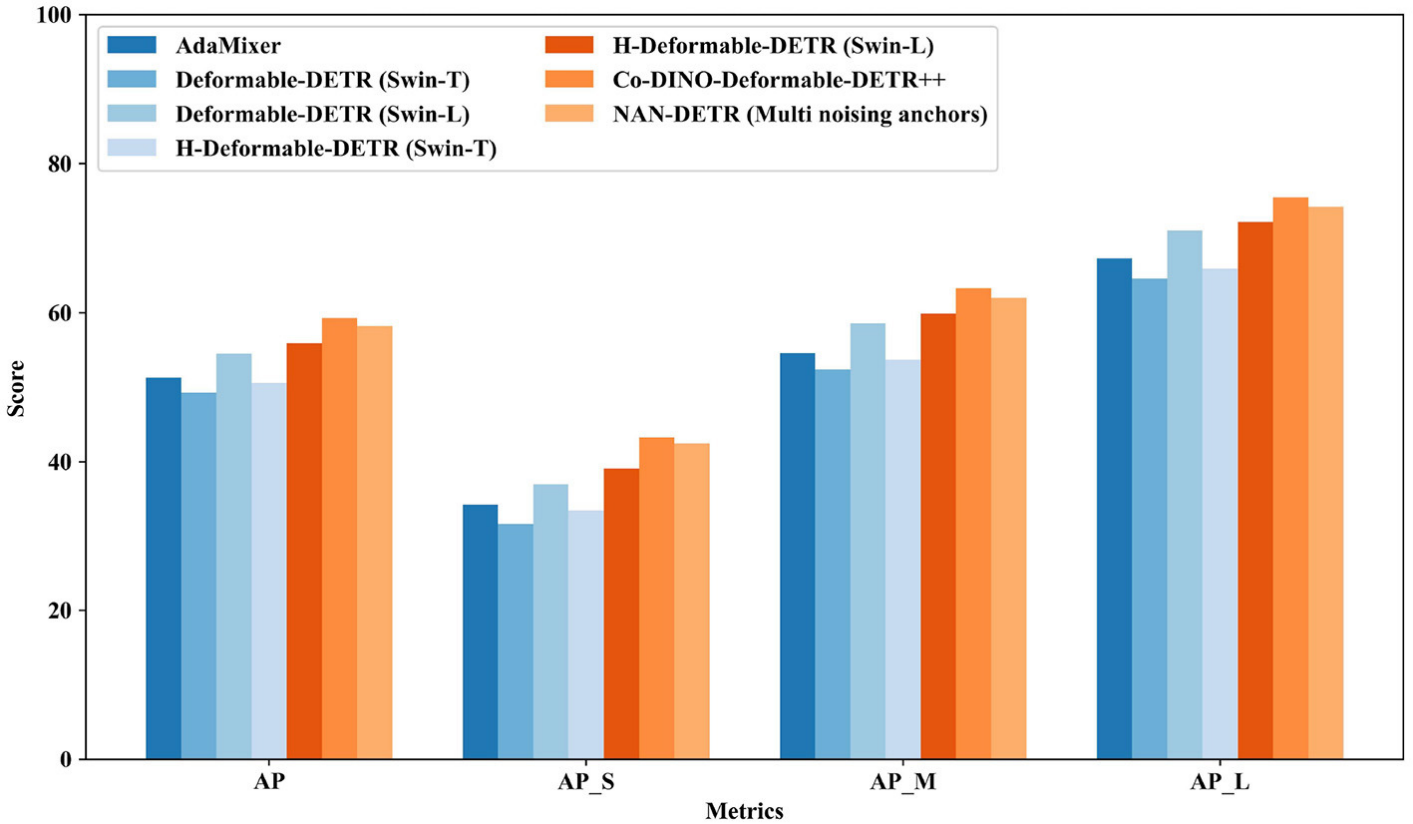
Comparison to the baselines DETR variants on COCO val with Swin.

The performance with the Swin backbone in Table 2 and Figure 6 shows considerable improvements, particularly for models utilizing advanced features of the Swin transformers. Methods like AdaMixer (Gao et al., 2022) with Swin-S backbone achieve an AP of 51.3%, showing good performance with adaptive mixing. Deformable-DETR (Zhu et al., 2021) with Swin-T and Swin-L backbones show significant improvements with AP scores of 49.3% and 54.5%, respectively, leveraging deformable attention modules to enhance detection accuracy.

**Table 2:** Comparison to the baselines on COCO val with Swin.

**Method**	**Backbone**	**Multi-scale**	**#query**	**#epochs**	**AP↑**	**AP_*S*_↑**	**AP_*M*_↑**	**AP_*L*_↑**
AdaMixer; Gao et al. (2022)	Swin-S	✓	300	36	51.3	34.2	54.6	67.3
Deformable-DETR; Zhu et al. (2021)	Swin-T	✓	300	12	49.3	31.6	52.4	64.6
Deformable-DETR; Zhu et al. (2021)	Swin-L	✓	300	12	54.5	37.0	58.6	71.0
H-Deformable-DETR; Jia et al. (2023)	Swin-T	✓	300	12	50.6	33.4	53.7	65.9
H-Deformable-DETR; Jia et al. (2023)	Swin-L	✓	300	12	55.9	39.1	59.9	72.2
Co-Deformable-DETR; Zong et al. (2023)	Swin-L	✓	900	36	**58.5**	42.4	**62.4**	74.0
NAN-DETR(CIoU, Multi-noising anchors)	Swin-L	✓	900	12	58.2	**42.5**	62.0	**74.2**

Bolded and underlined fonts indicate the first two best values.

H-Deformable-DETR (Jia et al., 2023) continues this trend with Swin-T and Swin-L backbones, achieving AP scores of 50.6% and 55.9%, respectively. Co-Deformable-DETR (Zong et al., 2023) with the Swin-L backbone achieves a strong AP of 58.5%, showcasing the effectiveness of collaborative hybrid assignments and the importance of detection heads. This method also achieves the highest AP_*M*_ of 62.4%, indicating superior performance in detecting medium-sized objects.

NAN-DETR with the Swin-L backbone, on the other hand, achieves an impressive AP of 58.2%, very close to Co-Deformable-DETR. However, it outperforms all other methods in the AP_*L*_ metric with a score of 74.2%, demonstrating exceptional performance in detecting large objects. This indicates that NAN-DETR's decoder-based multi-anchor strategy and centralization noising mechanism are highly effective for tasks requiring precise detection of larger objects.

These results demonstrate that both Co-Deformable-DETR and NAN-DETR excel in different aspects of object detection. Co-Deformable-DETR leads in medium-sized object detection, while NAN-DETR stands out in large object detection. The integration of the CIoU loss in NAN-DETR also enhances bounding box prediction accuracy, making both models valuable tools for a wide range of object detection scenarios.

#### 4.3.3 Overall analysis

**Figure 7:**
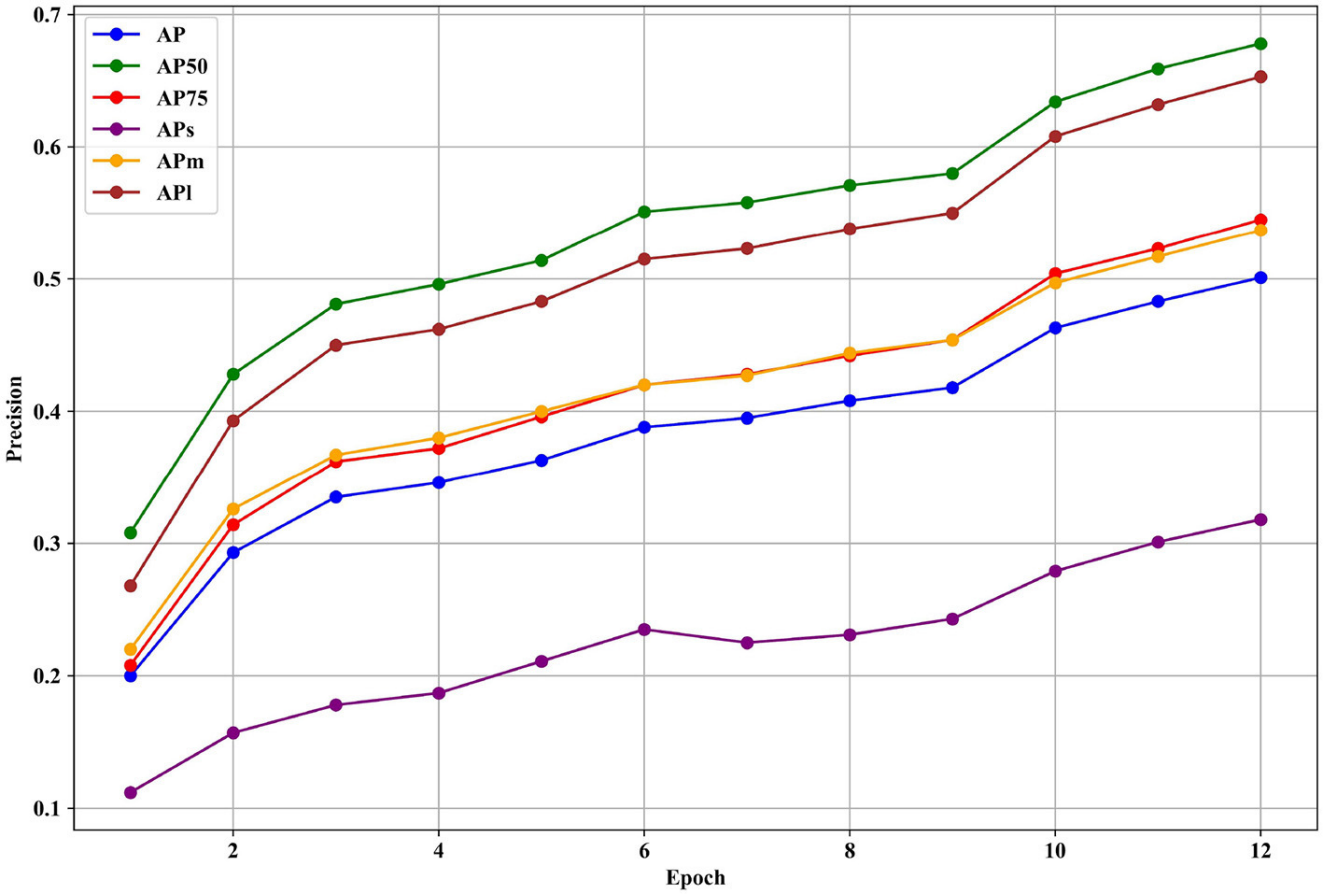
The various precision of NAN-DETR with CIoU and multi-noising anchors on COCO dataset. These detectors are trained with ResNet-50 backbones for 12 epochs.

Figure 7 presents the various precision metrics of the NAN-DETR model on the COCO dataset, specifically focusing on the effects of the CIoU loss and Multi-noising anchors. The figure illustrates how the NAN-DETR model performs in terms of Average Precision (AP) across different IoU thresholds and object sizes after being trained for 12 epochs using the ResNet-50 backbone.

**Analysis of Performance:**

- **AP (average precision) improvement:** the NAN-DETR model with the CIoU loss and multi-noising anchors achieves an AP of 50.1%, which is a significant improvement over other configurations, including the baseline NAN-DETR with single anchors and no CIoU. This suggests that the combination of CIoU and multi-noising anchors contributes to more accurate detection, especially for bounding box predictions.
- **Small object detection:** while the overall performance shows improvement, the precision for small objects (AP_*S*_) sees a slight decrease in the model with multi-noising anchors. The AP_*S*_ drops from 32.5% in the single-anchor configuration to 31.8% with multi-noising anchors. This could indicate that the centralization noising mechanism, while beneficial overall, may introduce challenges in detecting smaller objects where precise bounding box predictions are critical.
- **Medium and large object detection:** the model demonstrates robustness in detecting medium and large objects, with AP_*M*_ (AP for medium objects) and AP_*L*_ (AP for large objects) improving to 53.7% and 65.3%, respectively. The consistent improvement in these metrics highlights the effectiveness of the multi-noising approach in handling objects with varying sizes and aspect ratios.
- **Impact of CIoU loss:** the use of the CIoU loss appears to enhance bounding box accuracy significantly, particularly for larger objects. The CIoU takes into account not just the overlap but also the aspect ratio and center distance between boxes, leading to more precise predictions. This is reflected in the higher AP_75_ score, indicating that the model performs better at stricter IoU thresholds.

#### 4.3.4 Ablation study

##### 4.3.4.1 Decoder-based multi-anchor strategy

**Figure 8:**
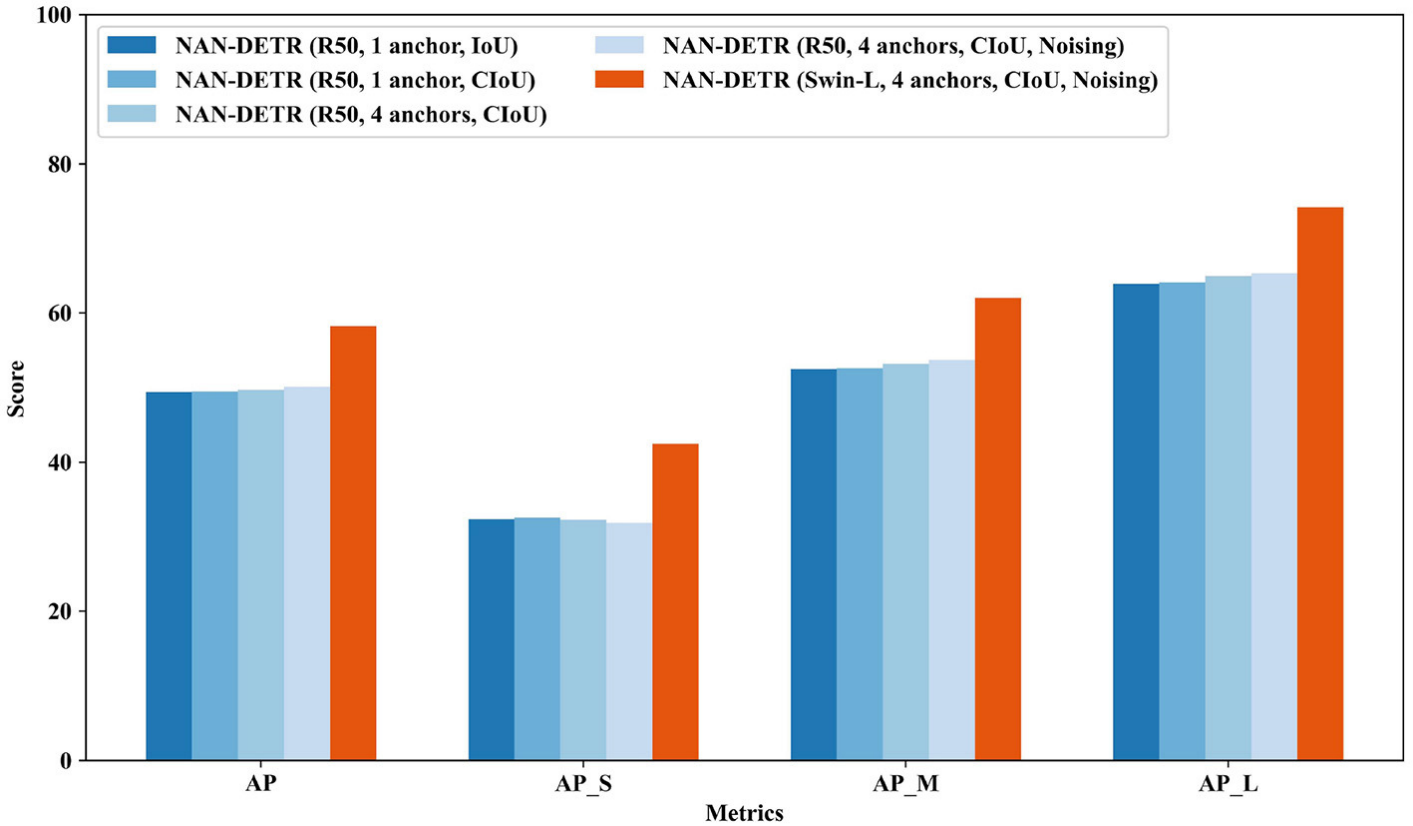
Ablation study on NAN-DETR on COCO val.

The ablation study from Table 3 and Figure 8 highlights the contributions of each component in NAN-DETR. Introducing multiple anchors without noising increases the AP to 49.7%, demonstrating the effectiveness of our decoder-based multi-anchor strategy in improving detection performance. The multiple anchors allow the model to better capture objects of varying sizes and shapes, leading to improved precision in detection tasks.

**Table 3:** Ablation study on NAN-DETR.

**Method**	**Backbone**	**Anchors**	**Noising**	**Box loss**	**AP↑**	**AP_*S*_↑**	**AP_*M*_↑**	**AP_*L*_↑**
NAN-DETR	R50	1	✗	IoU	49.4	32.3	52.5	63.9
NAN-DETR	R50	1	✗	CIoU	49.5	32.5	52.6	64.1
NAN-DETR	R50	4	✗	CIoU	49.7	32.2	53.2	64.9
NAN-DETR	R50	4	✓	CIoU	50.1	31.8	53.7	65.3
NAN-DETR	Swin-L	4	✓	CIoU	**58.2**	**42.5**	**62.0**	**74.2**

Bolded and underlined fonts indicate the first two best values.

##### 4.3.4.2 Centralization noising mechanism

When both multi-anchor and the centralization noising mechanism are combined, we see a significant jump in AP to 50.1%. This shows that the centralization noising mechanism, by introducing controlled randomness, enhances the stability of the model. As we anticipated, perturbing multiple anchor boxes toward the center greatly improves situations where the initial setup is overly divergent, making it difficult to achieve high precision. This perturbation enhances the model's stability to some extent. The AP increase across different object sizes (AP_*S*_, AP_*M*_, AP_*L*_) further confirms the benefits of these combined strategies in improving overall detection performance. The fact that AP_*M*_ and AP_*L*_ improve more quickly than AP_*S*_ further validates this idea. The larger the object, the more this perturbation reduces the loss caused by divergence. The decrease in AP_*S*_ is also understandable, likely due to an imbalance in weights caused by excessive duplication, which should be suppressed using NMS (Non-Maximum Suppression).

##### 4.3.4.3 Different backbone

From the perspective of changing the backbone, the Swin-L backbone indeed significantly outperforms smaller models like ResNet in terms of capturing global features, which is consistent with expectations. This observation leads to the idea of whether using a larger model as a backbone could be advantageous.

##### 4.3.4.4 Complete Intersection over Union (CIoU) loss

When comparing a single anchor with and without CIoU loss, we observe a slight improvement in AP from 49.4% to 49.5%, indicating that the CIoU loss provides a marginal but consistent boost in bounding box regression accuracy.

## 5 Conclusion

In this paper, we propose NAN-DETR, a novel object detection framework that integrates a decoder-based multi-anchor strategy, a centralization noising mechanism, and the Complete Intersection over Union loss. Our experimental results on the COCO dataset indicate that NAN-DETR delivers significantly improved detection accuracy compared to existing DETR variants. The multi-anchor strategy enhances the effectiveness of object matching, while the centralization noising mechanism and CIoU loss contribute to higher precision across various detection tasks.

Nonetheless, there is potential space for improvement in some aspects of the proposed approach. Currently, NAN-DETR does not prioritize processing speed or real-time performance, but a potential efficiency improvement strategy is to utilize model distillation techniques. On the other hand, a future direction is exploring to improve the centralization noising mechanism, possibly using learned parameters from the backbone network for dynamically adjusting the perturbation magnitude to further boost detection performance, particularly for small objects.

## Data Availability

Publicly available datasets were analyzed in this study. This data can be found here: http://images.cocodataset.org/zips/train2017.zip; http://images.cocodataset.org/zips/val2017.zip; http://images.cocodataset.org/annotations/annotations_trainval2017.zip.

## References

[B1] Bodla N. Singh B. Chellappa R. Davis L. S. (2017). “Soft-nms - improving object detection with one line of code,” in IEEE *International Conference on Computer Vision, ICCV 2017* (Venice: IEEE Computer Society), 5562–5570.

[B2] Carion N. Massa F. Synnaeve G. Usunier N. Kirillov A. Zagoruyko S. (2020). End-to-end object detection with transformers. arXiv [preprint] abs/2005.12872. 10.1007/978-3-030-58452-8_13

[B3] Chen Q. Chen X. Wang J. Zhang S. Yao K. Feng H.. (2023). “Group detr: Fast detr training with group-wise one-to-many assignment,” in Proceedings of the IEEE International Conference on Computer Vision (ICCV) (Paris: 2023).

[B4] Chen X. Wei F. Zeng G. Wang J. (2022). Conditional detr v2: Efficient detection transformer with box queries. arXiv [preprint] abs/2207.08914. 10.48550/arXiv.2207.08914

[B5] Chen Z. Zhang J. Tao D. (2022). “Recurrent glimpse-based decoder for detection with transformer,” in IEEE/CVF Conference on Computer Vision and Pattern Recognition, CVPR 2022 (New Orleans, LA: IEEE), 5250–5259.

[B6] Dai X. Chen Y. Yang J. Zhang P. Yuan L. Zhang L. (2021). “Dynamic DETR: end-to-end object detection with dynamic attention,” in 2021 IEEE/CVF International Conference on Computer Vision, ICCV 2021 (Montreal, QC: IEEE), 2968–2977.

[B7] Dai Z. Cai B. Lin Y. Chen J. (2022). Unsupervised pre-training for detection transformers. IEEE Trans. Pattern Analy. Mach. Intellig. 45, 12772–12782. 10.1109/TPAMI.2022.3216514 36269904

[B8] Gao P. Zheng M. Wang X. Dai J. Li H. (2021). “Fast convergence of DETR with spatially modulated co-attention,” in 2021 IEEE/CVF International Conference on Computer Vision, ICCV 2021 (Montreal, QC: IEEE).

[B9] Gao Z. Wang L. Han B. Guo S. (2022). “Adamixer: A fast-converging query-based object detector,” in IEEE/*CVF Conference on Computer Vision and Pattern Recognition, CVPR 2022* (New Orleans, LA: IEEE), 5354–5363.

[B10] Girshick R. B. Donahue J. Darrell T. Malik J. (2014). “Rich feature hierarchies for accurate object detection and semantic segmentation,” in 2014 IEEE Conference on Computer Vision and Pattern Recognition, CVPR 2014 (Columbus, OH: IEEE), 580–587.

[B11] He K. Zhang X. Ren S. Sun J. (2016). “Deep residual learning for image recognition,” in 2016 IEEE Conference on Computer Vision and Pattern Recognition, CVPR 2016 (Las Vegas, NV: IEEE), 770-778.

[B12] Hou X. Liu M. Zhang S. Wei P. Chen B. (2024). “Salience detr: Enhancing detection transformer with hierarchical salience filtering refinement,” in 2024 IEEE/CVF Conference on Computer Vision and Pattern Recognition (CVPR) (Seattle, WA), 17574–17583. 10.1109/CVPR52733.2024.01664430

[B13] Jia D. Yuan Y. He H. Wu X. Yu H. Lin W.. (2023). “Detrs with hybrid matching,” in IEEE/CVF Conference on Computer Vision and Pattern Recognition (CVPR) (Vancouver, BC: IEEE), 19702–19712. 10.1109/CVPR52729.2023.01887

[B14] Kong T. Sun F. Liu H. Jiang Y. Li L. Shi J. (2020). Foveabox: beyound anchor-based object detection. IEEE Trans. Image Proc. 29, 7389–7398. 10.1109/TIP.2020.3002345

[B15] Kuhn H. W. (1955). The hungarian method for the assignment problem. Naval Res. Logist. Quart. 2, 83–97. 10.1002/nav.3800020109

[B16] Li F. Zhang H. Liu S. Guo J. Ni L. M. Zhang L. (2022). “DN-DETR: accelerate DETR training by introducing query denoising,” in IEEE/CVF Conference on Computer Vision and Pattern Recognition, CVPR 2022 (New Orleans, LA: IEEE), 13609–13617. 10.1109/TPAMI.2023.3335410 38019624

[B17] Lin T.-Y. Maire M. Belongie S. Bourdev L. Girshick R. Hays J.. (2015). Microsoft coco: Common objects in context. arXiv [preprint] abs/1405.0312. 10.48550/arXiv.1405.0312

[B18] Liu F. Wei H. Zhao W. Li G. Peng J. Li Z. (2021). “WB-DETR: transformer-based detector without backbone,” in 2021 IEEE/CVF International Conference on Computer Vision, ICCV 2021 (Montreal, QC: IEEE), 2959–2967.

[B19] Liu S. Li F. Zhang H. Yang X. Qi X. Su H.. (2022). “DAB-DETR: dynamic anchor boxes are better queries for DETR,” in The Tenth International Conference on Learning Representations, ICLR 2022.

[B20] Liu Z. Hu H. Lin Y. Yao Z. Xie Z. Wei Y.. (2022). “Swin transformer V2: scaling up capacity and resolution,” in IEEE/CVF Conference on Computer Vision and Pattern Recognition, CVPR 2022 (New Orleans, LA: IEEE), 11999-12009.

[B21] Liu Z. Lin Y. Cao Y. Hu H. Wei Y. Zhang Z.. (2021). “Swin transformer: Hierarchical vision transformer using shifted windows,” in 2021 IEEE/CVF International Conference on Computer Vision, ICCV 2021 (Montreal, QC: IEEE), 9992–10002.

[B22] Meng D. Chen X. Fan Z. Zeng G. Li H. Yuan Y.. (2021). “Conditional DETR for fast training convergence,” in 2021 IEEE/CVF International Conference on Computer Vision, ICCV 2021 (Montreal, QC: IEEE), 3631–3640.

[B23] Ouyang-Zhang J. Cho J. H. Zhou X. Krähenbühl P. (2022). NMS strikes back. arXiv [preprint] abs/2212.06137. 10.48550/arXiv.2212.06137

[B24] Redmon J. Divvala S. K. Girshick R. B. Farhadi A. (2016). “You only look once: Unified, real-time object detection,” in 2016 IEEE Conference on Computer Vision and Pattern Recognition, CVPR 2016 (Las Vegas, NV: IEEE), 779–788.

[B25] Ren S. He K. Girshick R. B. Sun J. (2015). “Faster R-CNN: towards real-time object detection with region proposal networks,” in Advances in Neural Information Processing Systems 28: *Annual Conference on Neural Information Processing Systems 2015*, eds. C. Cortes, N. D. Lawrence, D. D. Lee, M. Sugiyama, and R. Garnett (Montreal, QC), 91–99.

[B26] Rezatofighi H. Tsoi N. Gwak J. Sadeghian A. Reid I. D. Savarese S. (2019). “Generalized intersection over union: A metric and a loss for bounding box regression,” in IEEE Conference on Computer Vision and Pattern Recognition, CVPR 2019 (Long Beach, CA: IEEE), 658–666.

[B27] Roh B. Shin J. Shin W. Kim S. (2022). “Sparse DETR: efficient end-to-end object detection with learnable sparsity,” in The Tenth International Conference on Learning Representations, ICLR 2022 (Virtual Event: OpenReview.net).

[B28] Vaswani A. Shazeer N. Parmar N. Uszkoreit J. Jones L. Gomez A. N.. (2017). “Attention is all you need,” in Advances in Neural Information Processing Systems 30: Annual Conference on Neural Information Processing Systems 2017, eds. I. Guyon, U. von Luxburg, S. Bengio, H. M. Wallach, R. Fergus, S. V. N. Vishwanathan, eds. (Long Beach, CA: IEEE), 5998–6008.

[B29] Wang Y. Zhang X. Yang T. Sun J. (2022). Anchor detr: query design for transformer-based detector. Proc. AAAI Conf. Artif. Intellig.36, 2567–2575. 10.1609/aaai.v36i3.20158

[B30] Yao Z. Ai J. Li B. Zhang C. (2021). Efficient DETR: improving end-to-end object detector with dense prior. arXiv [preprint] abs/2104.01318. 10.48550/arXiv.2104.01318

[B31] Zhang H. Li F. Liu S. Zhang L. Su H. Zhu J.. (2023). “DINO: DETR with improved denoising anchor boxes for end-to-end object detection,” in The Eleventh International Conference on Learning Representations (Kigali).

[B32] Zhang S. Chi C. Yao Y. Lei Z. Li S. Z. (2020). “Bridging the gap between anchor-based and anchor-free detection via adaptive training sample selection,” in 2020 IEEE/CVF Conference on Computer Vision and Pattern Recognition, CVPR 2020 (Seattle, WA: IEEE), 9756–9765.

[B33] Zhao Y. Lv W. Xu S. Wei J. Wang G. Dang Q.. (2024). “Detrs beat yolos on real-time object detection,” in Proceedings of the IEEE/CVF Conference on Computer Vision and Pattern Recognition (CVPR), 16965–16974.

[B34] Zheng Z. Wang P. Liu W. Li J. Ye R. Ren D. (2020). “Distance-iou loss: Faster and better learning for bounding box regression,” in The Thirty-Fourth AAAI Conference on Artificial Intelligence, AAAI 2020, The Thirty-Second Innovative Applications of Artificial Intelligence Conference, IAAI 2020, The Tenth AAAI Symposium on Educational Advances in Artificial Intelligence, EAAI 2020 (New York, NY: AAAI Press), 12993–13000.

[B35] Zhu X. Su W. Lu L. Li B. Wang X. Dai J. (2021). “Deformable DETR: deformable transformers for end-to-end object detection,” in 9th International Conference on Learning Representations, ICLR 2021 (Virtual Event: OpenReview.net).

[B36] Zong Z. Song G. Liu Y. (2023). “Detrs with collaborative hybrid assignments training,” in Proceedings of the IEEE/CVF International Conference on Computer Vision, 6748–6758.

